# Thermal Performance of School Buildings: Impacts beyond Thermal Comfort

**DOI:** 10.3390/ijerph19105811

**Published:** 2022-05-10

**Authors:** Bin Su, Renata Jadresin Milic, Peter McPherson, Lian Wu

**Affiliations:** 1 School of Architecture, Unitec Institute of Technology, Auckland 0600, New Zealand; rjadresinmilic@unitec.ac.nz (R.J.M.); pmcpherson@unitec.ac.nz (P.M.); 2 School of Healthcare and Social Practice, Unitec Institute of Technology, Auckland 0600, New Zealand; lwu@unitec.ac.nz

**Keywords:** building envelope, indirect health effects, indoor allergen, indoor microclimate, indoor thermal environment, insulation and thermal mass, school building, thermal comfort, occupant health, thermal performance

## Abstract

Based on field study data regarding the winter indoor thermal environment of three classrooms with different building envelopes, this study compared and evaluated these environments, not only related to students’ thermal comfort but also to their health. The inadequacy of the conventional New Zealand school building for maintaining a comfortable and healthy winter indoor thermal environment has been identified. A classroom with thermal mass had 31%, 34% and 9% more time than a classroom without thermal mass when indoor temperatures met 16 °C 18 °C and 20 °C respectively and has 21.4% more time than the classroom without thermal mass when indoor relative humidity was in the optimal range of 40% to 60%, in a temperate climate with a mild and humid winter. Adding thermal mass to school building envelopes should be considered as a strategy to improve the winter indoor thermal environment in future school design and development. Adding thermal mass to a school building with sufficient insulation can not only increase winter indoor mean air temperature but can also reduce the fluctuation of indoor air temperatures. This can significantly reduce the incidence of very low indoor temperature and very high indoor relative humidity, and significantly improve the indoor thermal environment.

## 1. Introduction

The World Health Organization recommends a minimum indoor temperature of 18 °C, and 20–21 °C for more vulnerable occupants, such as older people and young children [1,2]. Previous studies show that the minimum threshold of indoor temperature required for limiting respiratory infections is 16 °C, meaning that there is an increased risk of respiratory infections when indoor temperatures are below 16 °C. A growing body of epidemiological evidence shows links between indoor temperatures and excess winter mortality and morbidity in various European regions, although difficulties of demonstrating direct causality exist. To date, studies relating to cold homes and health effects have been largely carried out in the United Kingdom, Ireland and New Zealand. However, epidemiological research has shown that the problem of cold indoor temperatures is replicated in other countries. Where buildings are designed primarily to cope with extreme summer temperatures, houses may not effectively protect against low temperatures during the relatively brief but cold winter season [3,4]. Multidisciplinary perspectives have been applied, and the current research findings on cold, ill health, and energy use and the impact of low indoor temperature on occupants’ health include a range of brief reviews, case studies and policy analyses. Indoor temperatures below 12 °C can cause short-term increases in blood pressure and blood viscosity, which may increase winter morbidity and mortality due to heart attacks and strokes. When elderly people are exposed to indoor temperatures of 9 °C or below for two or more hours, their deep body temperature can start decreasing [5,6,7]. An extremely low indoor temperature not only negatively impacts occupants’ thermal comfort, but also occupants’ health. The indoor environment of school classrooms is mainly focused on thermal comfort, chemical air pollutants and microbiological stressors, which can impact and potentially affect students’ health [8]. The quality of the indoor thermal environment is very important for students’ health and performance, and the classroom should provide a conducive environment to promote teaching and learning [9,10,11,12].

There are a number of studies related to the thermal comfort of primary and secondary school classrooms in countries with different climates. Some include a literature review on thermal comfort aspects within schools, focusing on the effects of thermal quality on the students’ learning performance [13]. The thermal comfort and thermal comfort parameters for children in primary school classrooms in three different schools in the Netherlands have also been investigated [14]. Results from thermal comfort surveys done in eight primary schools in the West Midlands, UK, showed a direct link between the attainment of children at school and the thermal conditions in classrooms and suggested that simply designing to a threshold comfort temperature was not enough to ensure that the most effective learning environments are delivered [15]. Findings from thermal comfort surveys and measurements of indoor environmental variables in naturally ventilated classrooms in Hampshire, England, suggest that children are more sensitive to higher temperatures than adults, with comfort temperatures being about 4 °C and 2 °C lower than the PMV and the EN 15251 adaptive comfort model predictions, respectively [16]. Indoor environmental conditions examined in seven primary schools near Venice confirmed that studying in a comfortable environment enhances students’ well-being and satisfaction and, therefore, their productivity and learning [17]. A study investigating the effects of building envelope energy regulations on the thermal comfort level in naturally ventilated classrooms in primary and secondary schools in Taiwan confirmed that building envelope design has a remarkable impact on indoor thermal conditions in naturally ventilated spaces [18]. The results of a field study about indoor thermal comfort, based on investigations in Italian classrooms show a trend characterized by a gradual change in the thermal preference from the heating season to the mid and warm season [19]. Examination of the seasonal performance, occupants’ comfort and cold stress in cross-laminated timber school buildings in the USA specifically showed the impact of lower temperatures in different school spaces [20]. The existing knowledge in the field includes an investigation of the thermal comfort in non-air-conditioned schools, and proposes an expectancy factor value for the Mediterranean climate [21]. Moreover, the results of a field study of indoor thermal comfort, based on investigations in Portuguese secondary schools’ classrooms, consisted of measuring the environmental parameters: air temperature (Ta), air relative humidity (RH), and CO_2_ concentrations [22]. A field study was conducted in a secondary school building in Cyprus to assess the indoor thermal conditions during the students’ lesson hours (school hours); air temperature (AT) and relative humidity (RH) were monitored using indoor and outdoor sensors simultaneously throughout the four seasons of the year, and data analysis compared the results with international standards, ASHRAE Standard 55, ISO Standard 7730, etc. [23]. These previous studies provided valuable insight and informed this research about new cross-disciplinary approaches that have been developed, starting from existing standards, best practices, policies, guidelines, techniques, procedures, and tools at the international level. In all these previous studies, there seems to be no controversy about the importance of the thermal performance of school buildings. However, most of these studies have been concerned with energy efficiency rather than the relationship between the building envelope and indoor health. This study specifically considers this relationship and provides results on how different building envelopes impact the indoor health of school buildings. Information on the correlations between indoor environments, health and educational outcomes are sorely limited in New Zealand [24]. There are limited data and studies on measurements of the New Zealand school environment, especially for indoor thermal conditions and indoor air quality [25]. Based on field-study data of indoor thermal environments, this study investigates the impacts of classrooms with different building envelopes on the winter indoor air temperature, not only related to students’ thermal comfort but also to their health.

Auckland has a temperate climate with a comfortable warm, dry summer and mild, wet winter. Most of the factors that adversely affect health, such as bacteria, viruses, fungi, mites, respiratory rhinitis and asthma, and chemical interactions, increase in conditions of high indoor relative humidity. Maintaining the indoor relative humidity at between 40% and 60% can minimise indirect health effects [26]. New Zealand has some of the highest levels of indoor dust-mite allergens in the world [27]. The indoor relative humidity required by dust mites to thrive is 75–80% or higher, and dust mites prefer temperatures of around 18–25 °C [26,28,29,30,31,32]. Maintaining indoor relative humidity below 50% can reduce indoor dust mites and their allergens, and mite populations are almost eliminated in winter when indoor relative humidity is maintained within 40 to 50% [31,33,34]. Recent studies show that, in order to maintain indoor dust-mite allergens at an acceptable level, winter indoor mean relative humidity adjacent to the floor must be maintained below the threshold for dust mites to thrive [35,36]. According to international and national standards, indoor relative humidity should be lower than 60% for optimum indoor air quality [37,38,39].

Visible mould growth on indoor surfaces is a common problem in over 30% of New Zealand houses [40]. The threshold of indoor relative humidity for mould survival and growth conditions is 60%. Mould growth is likely on almost any building material if equilibrium relative humidity of the material exceeds 75–80% [41,42,43]. Mould germination requires not only high relative humidity (80%), but also time (30 days) [44]. One option to prevent mould growth on indoor surfaces is to control the indoor relative humidity to a level below the threshold (80%) of mould germination [9,45].

In New Zealand, students normally stay in school for about six hours a day, and 90% of classrooms are designed for natural ventilation using windows [46]. In winter, when windows of school buildings are typically closed, the air change rate in classrooms is much lower than the requirement of New Zealand standards [47]; consequently, indoor CO_2_ concentration often exceeds the guideline value [46,48,49,50]. Indoor air pollutants in a classroom can have serious and long-lasting negative impacts on the students. There are significant studies on health-related exposures in New Zealand and overseas schools. Some of the studies analysed the concentration and sources of air pollution in a school in New Zealand to understand the factors critical for assessment and to develop strategies for controlling and reducing exposure to indoor air pollution [51]. Similarly, research with an analysis and assessment of the indoor air quality of 27 primary schools in Belgium aimed to obtain correlations between the various pollutant levels concerning the comfort and health of the students [52]. Other studies identified that, while respiratory health effects of damp housing are well recognised, less is known about the impact of dampness and water damage in schools, when developing a study on the correlation between school dampness, levels of fungal and bacterial markers, respiratory symptoms and lung function, in children in schools in Spain, the Netherlands and Finland [53]. To assess school buildings’ characteristics in relation to the students’ comfort and health, existing literature examined the concurrent exposures of young children to past-use and current-use pesticides in their everyday environments [54]. Similarly, some of the research on the relation between exposure to ambient total fungal spores and a reduction of childhood lung function extended to determine what specific fungal spores were responsible for observed changes in lung function in schoolchildren in Taiwan [55]. Others conducted a health-risk assessment for chronic toxic effects and cancer and measured concentrations of volatile organic compounds (VOCs) in the classrooms, kindergartens, and outdoor playgrounds of three primary schools in the spring, winter, and fall terms in Turkey [56]. Indoor air pollutants and health effects in New Zealand schools have not been extensively investigated [24]. Auckland has a humid winter, and major indoor air pollutants and health effects such as bacteria, viruses, fungi, mites, respiratory rhinitis and asthma, and chemical interactions are closely related to indoor high relative humidity [26]. This study also investigates the impact of classrooms with different envelopes on winter indoor relative humidity levels, which can impact major local indirect health effects of relative humidity, such as mould and dust-mites.

**Figure 1:**
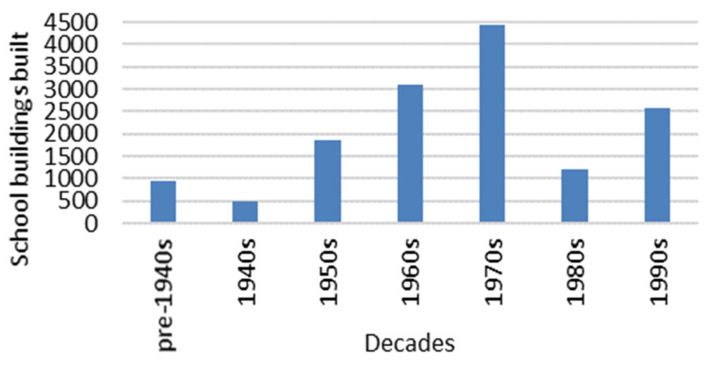
Number of school buildings built from pre-1940s to 1990s in New Zealand (source: the data was provided by the New Zealand Ministry of Education).

In New Zealand there are 14,637 school buildings built from the pre-1940s to the 1990s (see Figure 1). In accordance with the current building codes [38,48], there could be a significant number of New Zealand school classrooms without sufficient insulation in their envelopes and with single-glazed windows, which can negatively impact the indoor thermal environment. There were about 425 schools built in Auckland before 2010. An Auckland school commonly comprises a number of low-rise, isolated buildings spread over a large site. Most Auckland school buildings, with one to four classrooms in a row, have a large external surface area which includes two or three sides consisting of external walls and roof surface areas. For this type of school building, the building envelope becomes the most important factor determining the winter indoor thermal environment. Based on the field study, this study contributed physical data on the winter indoor thermal environment of three classrooms with different insulation and with or without thermal mass in their building envelopes in Auckland. This study also compared and evaluated thermal performance of school buildings with different envelopes under a temperate climate with mild and wet winter, which can be used in the processes of thermo-modernization of school buildings in New Zealand or overseas under similar climatic conditions.

## 2. Methods

The redevelopment of Avondale College, in West Auckland, from 2010 to 2014, represented one of the biggest school rebuilding programs in New Zealand’s history. The project included 92 new and refurbished teaching and resource spaces. Refurbished buildings in Avondale College are of conventional lightweight timber frame construction (timber structure) with internal insulation and lightweight external cladding. In the retrofitted school buildings at Avondale College, the original timber structures were retained, and the new building envelopes included lightweight walls and roofs with sufficient insulation and double-glazed windows. For the retrofitted school building, the project mainly focused on increasing the R-value in building envelopes without considering the thermal mass effect (i.e., without adding thermal mass in the building envelope). For some new school buildings in the Avondale College redevelopment project, insulated precast panels (with thermal mass) were used for the main structure and building envelope. This was the first time that insulated precast concrete panels had been used as the main structure and building envelope for new secondary-school buildings in New Zealand. It was also the first time for a school building with thermal mass in its envelopes to be used for a field study of the winter indoor thermal environment in New Zealand. This study compared and investigated differences in impact of school buildings with different building envelopes on the winter indoor thermal environment in a temperate climate with a mild and humid winter. 

**Figure 2:**
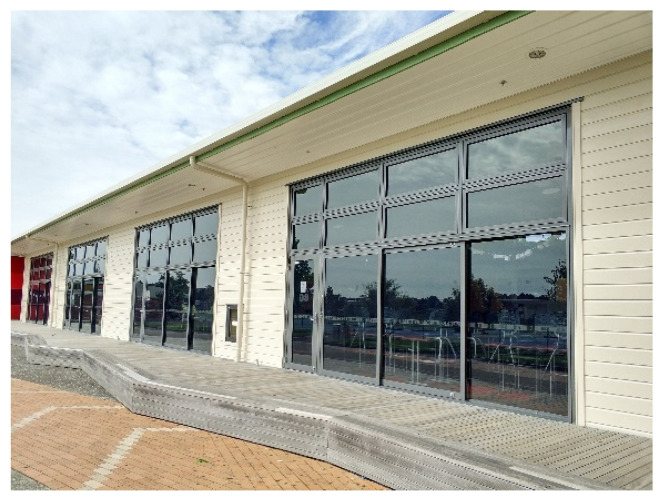
Retrofit school building.

**Figure 3:**
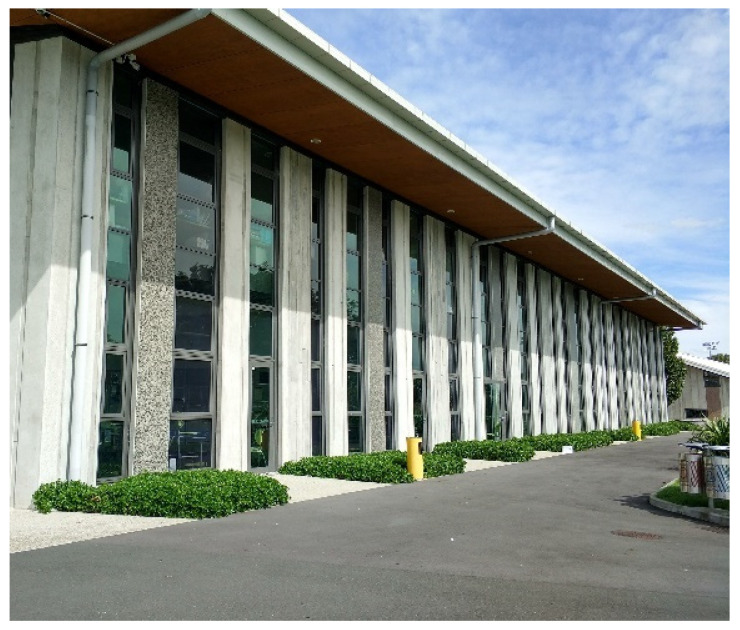
New school building with precast insulated concrete panel wall.

Three classrooms at Avondale College, with different R-values and with or without thermal mass in their building envelopes, were selected for the field study of winter indoor thermal environments. Classroom 1 is in an old one-storey prefab school building (built in the 1990s) without thermal mass and without sufficient insulation in its envelope (demolished after the field study) and with north orientation. Classroom 2 is in the middle of a newly retrofitted one-storey building (built in the 1990s and retrofitted in 2011) with sufficient insulation, double-glazed windows and without thermal mass in its envelope and with north orientation (see Figure 2), and has roof, north wall and south wall as its external envelope and a north orientation. The adjacent indoor spaces of Classroom 2 have the same space heating. Classroom 3 is in the middle and second floor of a new two-storey building (built in 2011) with sufficient insulation, double-glazed windows and thermal mass (precast insulated concrete panel wall and concrete structure) in its envelope and with north orientation (see Figure 3), and it has roof, north wall and south wall as its external envelope and a north orientation. The adjacent indoor spaces of Classroom 3 have the same space heating. Table 1 shows the construction elements of the three classrooms. The design data in Table 1 are derived from the building plans provided by Jasmax, the project’s architects. The study compares and evaluates winter indoor thermal conditions not only related to students’ thermal comfort but also to their health.

The field study of the winter indoor thermal environment of three classrooms with different R-values and with or without thermal mass in their building envelopes was carried out by the author at Avondale College during the winter months from 13 June to 22 September 2013. Air temperatures and relative humidity adjacent to the ceiling and the floor, and in the shaded outdoor spaces under the roof eaves of the three classrooms, were continuously measured at 15-min intervals, 24 h a day by a Lascar EL-USB-2 USB Humidity Data Logger. The measuring points adjacent to the ceiling and the floor were located close to the south internal walls and the measuring points under the roof eaves were on the south side of classrooms, which can minimize the impact of solar gain during the daytime. 

During the school hours, each sample classroom could be used for different courses, which could accommodate about 25 students. As the occupancy and use time for each classroom during the field study could be different, it is difficult to monitor or account for the heat gain of students for this study. The windows of the three classrooms were closed during the field study, and ratios of glazing surface to external wall area of Classroom 2 (retrofit) and Classroom 3 (thermal mass) are 0.4 and 0.41, respectively. (Classroom 1 was a very old building without a sufficient building plan and was demolished soon after the field study). There was no mechanical ventilation in the three classrooms during school hours at the time of the field study. The school buildings only used space heating during the school hours (from 8:30 a.m. to 3:30 p.m.), which was provided by the gas-boiler central-heating system. During the school hours, the indoor thermal environments of the sample classrooms are mainly impacted and controlled by space heating. This study not only investigated and compared the indoor thermal environments of school buildings with or without thermal mass during the school hours with the impact of space heating, but also during the night-time without the impact of space heating. During the school hours under the same space heating method, the field study data of the indoor thermal environments can be used to compare how the different building envelopes respond to space heating, especially for building envelopes with or without thermal mass. After school hours, especially during the night-time without the impact of space heating and the heat gain of students, the indoor temperatures could be significantly lower than during school hours and indoor relative humidity could be significantly higher than during school hours, which mainly depends on building thermal performance. As the local major indirect health effects of relative humidity such as mould and dust-mites increase in conditions of high indoor relative humidity, this study not only investigated and compared school buildings’ thermal performance and the indoor thermal environments of the three classrooms during the school hours and during the whole winter, but also during the night-time when the indoor thermal environment was not impacted by space heating and the heat gain of students.

The field-study data on air temperatures and relative humidity of indoor and outdoor environments have been converted into percentages of time related to different ranges of indoor air temperature and relative humidity throughout winter (24 h per day), winter night (from 7:00 p.m. to 7:00 a.m.) and winter school hours (from 8:30 a.m. to 3:30 p.m.), which can be used to compare the indoor thermal environment related to students’ thermal comfort and health. In accordance with WHO [1,2] and previous studies [3,4,5,6,7], the study used percentages of time when indoor air temperatures were greater than or equal to 16 °C, 18 °C, 20 °C and 22 °C to compare the indoor thermal environments related to students’ thermal comfort, and used percentages of time when indoor air temperatures were lower than 16 °C, 12 °C, 10 °C and 9 °C to compare indoor thermal conditions related to students’ health. The study also used indoor mean relative humidity and percentages of time when indoor relative humidity was in the optimal range of 40% to 60% [26] to compare indoor relative humidity levels related to students’ health. 

All field-study data have been converted into hourly mean temperature and relative humidity, which can be used to represent a mean variation of indoor temperature and relative humidity throughout the winter. The hourly mean temperature or relative humidity was derived from averaging all temperature or relative humidity data within a particular hour (e.g., at 1 a.m., 1:15 a.m., 1:30 a.m. and 1:45 a.m.) for all winter days. The hourly mean temperature and relative humidity data used in this study are the averages of the hundreds of temperature and relative humidity measurements within a particular hour on all winter days. As the temperature and relative humidity at a particular testing time on different winter days could be significantly different, the hourly mean temperatures and the hourly mean relative humidity for the whole winter may not precisely follow the correlation between air temperature and relative humidity (relative humidity decreases or increases in association with increasing or decreasing temperature), but ranges of their variations can still be identified and used to compare the indoor thermal environments. 

## 3. Data Analysis

### 3.1. Indoor Air Temperature

Throughout the winter (24 h per day), although the winter indoor mean air temperature of Classroom 3 (thermal Mass) was only 1.7 °C higher than Classroom 2 (retrofit) (see Table 2), Classroom 3 (thermal Mass) had 34% more time than Classroom 2 (retrofit) at indoor air temperatures greater than or equal to 18 °C, the minimum indoor air temperature for healthy thermal conditions required by the WHO [1]. Classroom 3 (thermal Mass) had 31% more time than Classroom 2 (retrofit) at indoor air temperatures greater than or equal to 16 °C, the minimum threshold of indoor temperature required for limiting respiratory infections [3,4]. The winter indoor mean air temperature of Classroom 2 (retrofit) with sufficient insulation was 2.4 °C higher than that of Classroom 1 (prefab) with insufficient insulation. Classroom 2 (retrofit) had 25% and 26% more time than Classroom 1 (prefab) at indoor air temperatures greater than or equal to 18 °C and 16 °C, respectively. Some New Zealand school classrooms without sufficient insulation in their envelopes and with single-glazed windows can have a poor winter indoor thermal environment related to students’ thermal comfort. With the same space-heating method, used during school hours only, Classroom 3 with thermal mass in its envelope had a significantly better winter indoor thermal environment related to students’ thermal comfort than Classroom 2 without thermal mass in its envelope.

During winter school hours (8:30 a.m. to 3:30 p.m.), the indoor thermal environment was mainly controlled by space heating. With the same space-heating method and duration, indoor mean air temperatures of Classroom 3 (thermal Mass) and Classroom 2 (retrofit) were very close; there was only 0.2 °C difference (see Table 3). Classroom 3 (thermal Mass) had 10% and 8% more time than Classroom 2 (retrofit) at indoor air temperatures greater than or equal to 18 °C and 16 °C, respectively. The winter indoor thermal environment of Classroom 3 (thermal Mass) was still better than that of Classroom 2 (retrofit) during school hours with the same space-heating method and duration. For 89% of winter school hours, the distribution of indoor air temperatures of Classroom 3 (thermal Mass) is between 16 °C to 22 °C, which is much more stable than Classroom 2 (retrofit), and the indoor mean maximum temperature is significantly lower Classroom 2 (retrofit). For 84% of winter school hours, the distribution of indoor air temperatures of Classroom 2 (retrofit) is between 16 °C to 26 °C, which could potentially cause over-heating problems. As Classroom 2 (retrofit) has lightweight building envelopes and internal insulation, the indoor space can be easily and quickly heated up and the indoor temperature can be very high (the mean maximum air temperature was up to 27.4 °C), which can negatively impact students’ thermal comfort. During school hours, the indoor thermal conditions of Classroom 3 (thermal Mass) had a stable and better indoor thermal environment related to students’ thermal comfort. The winter indoor mean temperature of Classroom 2 (retrofit) with sufficient insulation was 3.6 °C higher than that of Classroom 1 (prefab) with insufficient insulation. Classroom 2 (retrofit) had 43% and 34% more time than Classroom 1 (prefab) at indoor air temperatures greater than or equal to 18 °C and 16 °C, respectively.

After school hours the space heating was turned off, and the indoor mean air temperature of Classroom 2 (retrofit) experienced a big drop from 19.4 °C to 15.8 °C (see Table 3 and Table 4), whereas the indoor mean air temperature of Classroom 3 (thermal Mass) saw a small drop from 19.6 °C to 18.6 °C (see Table 3 and Table 4). During winter nights (7 p.m. to 7 a.m.) without space heating, the winter indoor mean air temperature of Classroom 3 (thermal Mass) was 2.8 °C higher than Classroom 2 (retrofit) (see Table 4). Classroom 3 (thermal Mass) had 54% and 47% more time than Classroom 2 (retrofit) at indoor air temperatures greater than or equal to 18 °C and 16 °C, respectively. Classroom 3 (thermal Mass) in its envelope had better indoor thermal conditions than Classroom 2 (retrofit) during winter nights (7 p.m. to 7 a.m.) under the local climate and with the current school building space heating method (limited permanent space-heating only during school hours). Classroom 3 (thermal mass) had 47% less time than Classroom 2 (retrofit) when indoor mean temperatures were lower than 16 °C. As there is an increased risk of respiratory infections when indoor temperatures are below 16 °C [3,4], Classroom 3 with thermal mass potentially had a better indoor thermal environment related to students’ health than Classroom 2 (retrofit) without thermal mass. The winter indoor mean air temperature of Classroom 2 (retrofit) with sufficient insulation was 1.7 °C higher than Classroom 1 (prefab) with insufficient insulation. Classroom 2 (retrofit) had 11% and 23% more time than Classroom 1 (prefab) at indoor air temperatures greater than or equal to 18 °C and 16 °C, respectively. Adding or increasing insulation in a school building envelope and using double glazing windows can significantly improve the winter indoor thermal environment. 

Throughout winter (24 h per day), Classroom 2 (retrofit) had 31%, 15% and 3% more time than Classroom 3 (thermal Mass) at indoor air temperatures lower than 16 °C, 14 °C and 12 °C, respectively (see Table 5). Classroom 1 (prefab) without sufficient insulation had 26%, 21%, 14%, 6% and 2% more time than Classroom 2 (retrofit) with sufficient insulation at indoor air temperatures lower than 16 °C, 14 °C, 12 °C, 10 °C and 9 °C, respectively. These very low temperatures usually occurred during the night and early morning, which can not only negatively impact students’ thermal comfort but also their health [3,4,5,6,7]. The very low indoor air temperatures can also result in very high indoor relative humidity, which can promote indirect health effects of relative humidity such as bacteria, viruses, fungi, mites, respiratory rhinitis and asthma, and chemical interactions [26].

### 3.2. Indoor Relative Humidity and Indoor Health Conditions

Throughout winter (24 h per day), indoor mean relative humidity of Classroom 3 (thermal Mass) (58%) and Classroom 2 (retrofit) (60%) were very close, and lower than or equal to 60%, with only a 2% difference (see Table 6). One of the major differences is that Classroom 3 (thermal Mass) had 21.4% more time than Classroom 2 (retrofit) when indoor relative humidity was in the range of 40% to 60%, which is the optimal range of relative humidity for indoor air quality and for minimising the indirect health effects of relative humidity. Other differences between Classroom 3 (thermal Mass) and Classroom 2 (retrofit) are the percentages of winter time at indoor relative humidity greater than or equal to 60%, 75% and 80%, respectively. 60% of relative humidity is the threshold for mould survival and growth conditions, 75–80% of relative humidity is required for dust mites to thrive and 80% of relative humidity is the threshold for mould germination [41,42,43,44]. Classroom 3 (thermal Mass) had apparently less time than Classroom 2 (retrofit) at indoor relative humidity greater than or equal to 60%, 75% and 80%, respectively. Under the local climate and the current space-heating method and duration, Classroom 3 with thermal mass and sufficient insulation had better indoor thermal environment related to students’ health, compared to the conventional Classroom 2 without thermal mass and with sufficient insulation. The winter indoor mean relative humidity (60%) of Classroom 2 (retrofit) was significantly lower than that of Classroom 1 (prefab) (70%). Classroom 2 (retrofit) had 43.3% more time than Classroom 1 (prefab) when indoor relative humidity was in the range of 40% to 60%. Classroom 2 (retrofit) had significantly less time than Classroom 1 (prefab) at indoor relative humidity greater than or equal to 60%, 75% and 80%, respectively. Sufficient insulation in the school building envelope can significantly improve the indoor thermal environment related to students’ health.

### 3.3. Fluctuation and Variation of Indoor Air Temperature and Relative Humidity

**Figure 4:**
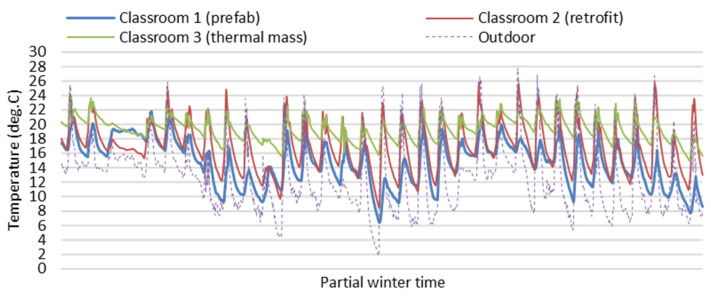
Field study data of indoor temperatures of the three classrooms on partial winter days (24 h per day).

**Figure 5:**
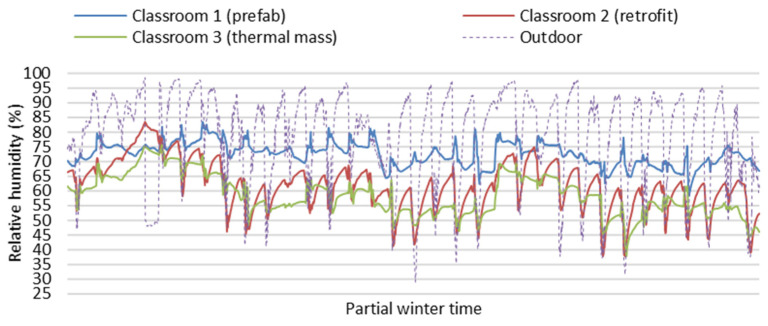
Field study data of indoor relative humidity of the three classrooms on partial winter days (24 h per day).

Figure 4 and Figure 5 show partial field study data of indoor temperatures and relative humidity of the three classrooms in the partial winter days from 13 June to 15 July (24 h per day). Indoor air temperatures of Classroom 3 with thermal mass were more stable and higher on average than Classroom 1 (prefab) and Classroom 2 (retrofit) without thermal mass during the winter (24 h per day) with the impact of space heating only during school hours in winter. Indoor relative humidity data of Classroom 3 (thermal Mass) were more stable and lower on average than Classroom 1 (prefab) and Classroom 2 (retrofit), as indoor relative humidity decreases associated with increasing air temperature. The large fluctuations of indoor air temperature in Classroom 1 (prefab) and Classroom 2 (retrofit) can result in very low indoor temperatures and very high relative humidity, which can negatively impact the winter indoor thermal environment related to students’ thermal comfort and health. Generally, Classroom 3 (thermal Mass) had a stable and better indoor thermal environment than Classroom 1 (prefab) and Classroom 2 (retrofit) during the winter.

**Figure 6:**
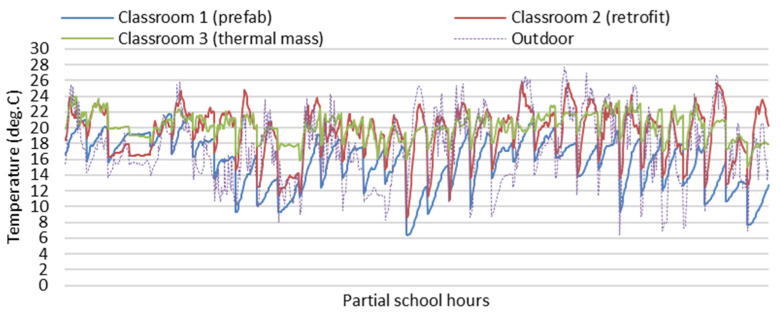
Field study data of indoor temperature of the three classrooms during school hours (8:30 a.m. to 3:30 p.m.).

**Figure 7:**
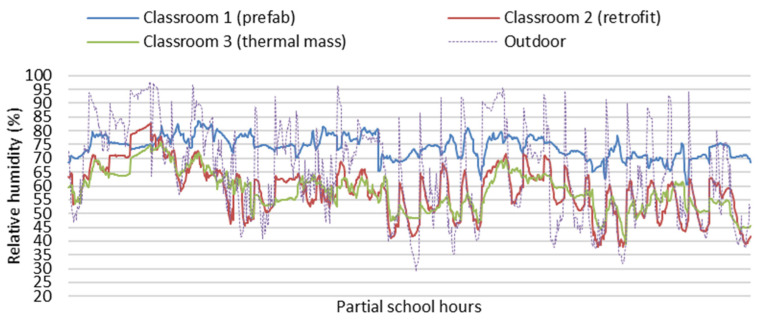
Field study data of indoor relative humidity of the three classrooms during school hours (8:30 a.m. to 3:30 p.m.).

Figure 6 and Figure 7 show partial field study data of indoor temperatures and relative humidity of the three classrooms during school hours (8:30 a.m. to 3:30 p.m.) on the partial winter days from 13 June to 15 July. As the field study data of indoor temperatures of the three classrooms were mainly impacted and controlled by the space heating during the school hours, it is hard to use this field study to identify the impacts of different building envelopes on the indoor thermal environment, and it is difficult to tell the difference in indoor temperature and relative humidity between the classrooms, especially for Classroom 2 and Classroom 3 with or without thermal mass.

**Figure 8:**
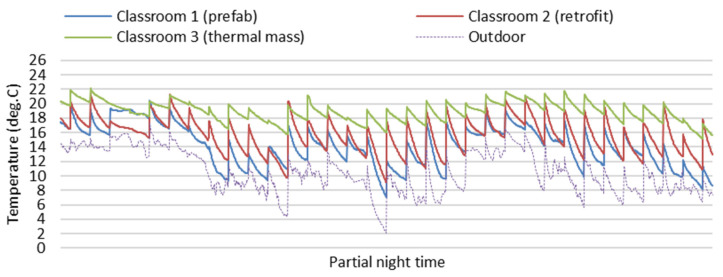
Field study data of indoor temperature of the three classrooms during winter nights (7 p.m. to 7 a.m.).

**Figure 9:**
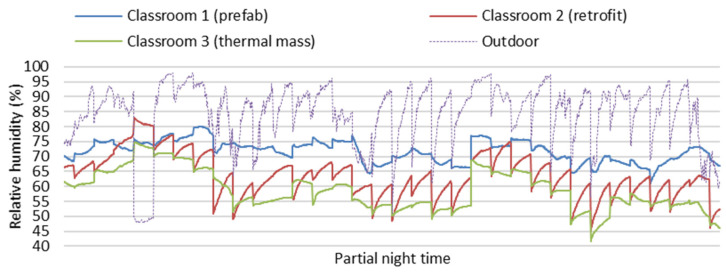
Field study data of indoor relative humidity of the three classrooms during winter nights (7 p.m. to 7 a.m.).

Figure 8 and Figure 9 show partial field study data of indoor temperatures and relative humidity of the three classrooms during winter nights (7 p.m. to 7 a.m.) on the partial winter days from 13 June to 15 July. During winter nights, without the impact of space heating and students’ heat gain, the indoor temperature and relative humidity of the three classrooms were mainly impacted by thermal performance of different building envelopes. Indoor air temperatures of Classroom 3 (thermal mass) were more stable and obviously higher than in Classroom 2 (retrofit), and indoor relative humidity in Classroom 3 (thermal Mass) was more stable and clearly lower than in Classroom 2 (retrofit). The winter thermal performance of Classroom 3 with thermal mass is better than Classroom 2 (retrofit) without thermal mass. 

**Figure 10:**
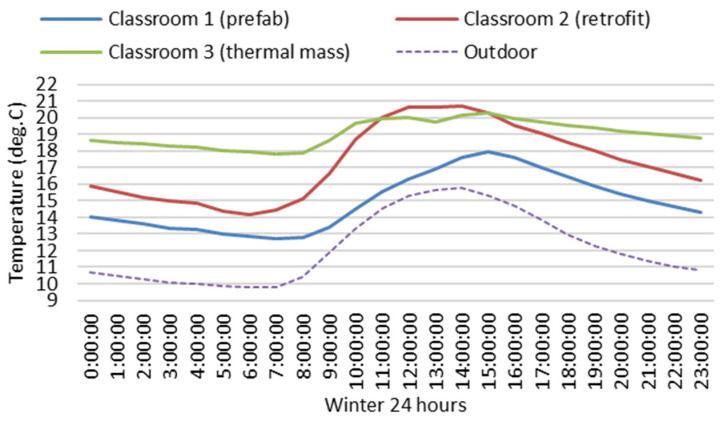
Winter indoor and outdoor hourly mean air temperatures of the three classrooms.

**Figure 11:**
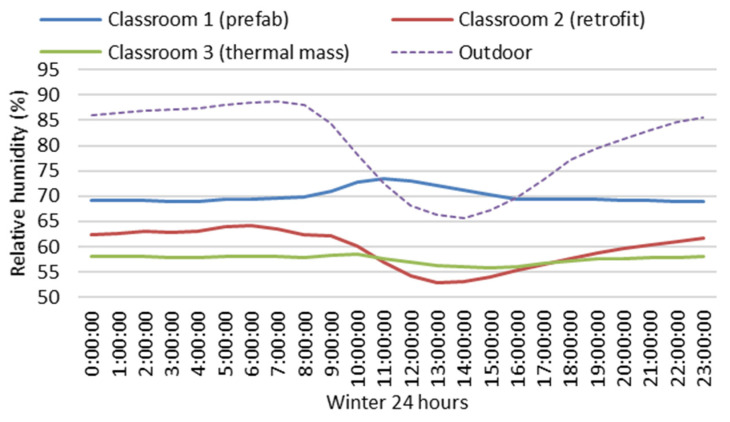
Winter outdoor and indoor hourly mean relative humidity of the three classrooms.

Figure 10 and Figure 11 show variations of winter indoor hourly mean air temperatures and relative humidity of the three classrooms. Throughout winter, indoor hourly mean air temperatures of Classroom 3 (thermal mass) were always higher than 18 °C or near 18 °C (the minimum hourly mean temperature is 17.8 °C) for 24 h. Indoor hourly mean air temperatures of Classroom 3 with thermal mass were much more stable and significantly higher than Classroom 2 without thermal mass during the late afternoon, evening, night and early morning. There were seven hours of space heating during the school day (from 8:30 a.m. to 3:30 p.m.). When indoor heat was lost through the building envelope, in Classroom 3 (thermal mass), some heat would be stored in the thermal mass in the building envelope with sufficient insulation, and then released back into the indoor space late after school hours, which would continuously maintain the indoor air temperature at a relative high level during the evening, night and early morning, which is good for the indoor thermal environment related to students’ thermal comfort and health, and potentially good for the energy efficiency of space heating. 

Indoor hourly mean air temperatures of Classroom 2 (retrofit) with sufficient insulation were significantly higher than those of Classroom 1 (prefab) with insufficient insulation. Indoor hourly mean air temperatures of Classroom 2 (retrofit) were lower than 18 °C for about 16 h before 10 a.m. and after 6 p.m. The minimum hourly mean air temperature of Classroom 2 (retrofit) (14.2 °C) was significantly lower than that of Classroom 3 (thermal Mass) (17.8 °C) in the early morning. The increase in indoor hourly mean air temperature of Classroom 2 (retrofit) from 14.1 °C (at 6 a.m.) to 18 °C (at 10 a.m.) took over four hours, which not only negatively impacts students’ thermal comfort and health but also potentially costs more space-heating energy in comparison to Classroom 3 (thermal mass).

Winter indoor hourly mean relative humidity of Classroom 3 (thermal mass) was more stable and lower than the other two classrooms without thermal mass. When winter indoor hourly mean temperatures of Classroom 3 (thermal mass) were generally in the healthy range of 17.8 °C and 20.3 °C (see Figure 10), indoor hourly mean relative humidity was also in the healthy range of 55.7% and 58.6% (see Figure 11) and always between 50% and 60%, the optimal range for relative humidity for indoor air quality, minimising the indirect health effects of relative humidity. When indoor hourly mean temperatures of Classroom 2 (retrofit) were lower than 18 °C during the night and early morning (see Figure 10), the indoor hourly mean relative humidity was higher than 60% (see Figure 11). Throughout winter, indoor hourly mean temperatures of Classroom 1 (prefab) were always and significantly lower than 18 °C (see Figure 10), and indoor hourly mean relative humidity was always higher than 60% (69% to 72.5%) (see Figure 11). Without sufficient insulation, the very low indoor air temperatures of Classroom 1 (prefab) resulted in very high indoor relative humidity, which can promote indirect health effects of relative humidity and negatively impact students’ health.

## 4. Discussion

For an Auckland low-rise and small size school building, thermal performance of the building envelope is crucial for indoor thermal and health conditions. The study provides first-hand field-study data and physical evidence to identify differences in the indoor thermal environment of school buildings with different building envelopes in the local climate with a mild and humid winter. It is the first time that the winter indoor thermal environment of a school building using insulated precast concrete panels as its main structure and building envelope has been investigated in New Zealand, which could influence conventional school building design and improve schools’ winter indoor thermal environment. According to the field study data, a school building with thermal mass in its envelope has significantly better winter indoor thermal and health conditions than a school building with a similar insulation level, but without thermal mass in its envelope, under limited permanent space-heating only during school hours. Findings of this study can draw architects’ and developers’ attention to the relationship between indoor environment quality and school building envelope design for the local climate. The findings can be used as a general guide or strategy for retrofitting old school buildings or for new school building development. The study was focused on classrooms in a school in Auckland, New Zealand, but the findings can be applicable to school building design in a temperate climate with a mild and humid winter. If the findings are applied, this will lead to improvement of schools’ indoor environmental quality related to students’ thermal comfort and health. 

A limitation of this study is the lack of information regarding comparison of field study data in summer. Although the mass effect can positively impact indoor thermal environment for both winter and summer, field study data during the summer are needed to prove that the school building with thermal mass in its envelope is adequate in the local climate. The monthly mean maximum temperatures during the summer are in the comfort zone, but the sun radiation during the summer months is quite high. A further research question is whether a school building with thermal mass in its building structure and envelope can positively impact the summer indoor thermal environment. Further field study during the summer can be used to compare and evaluate the thermal performance of school buildings with or without thermal mass in their envelopes in a temperate climate with warm and dry summer. Another limitation of this study is the lack of survey data on students’ thermal comfort and health. During the school term, the sample classrooms could be used for different courses or by different groups of students; it is difficult to identify the appropriate participants (students) for the survey related to the sample classrooms.

## 5. Conclusions

Based on the field-study data of Classroom 1 (prefab) in Table 2, Table 3 and Table 6, an old, conventional New Zealand school building with a timber structure, lightweight envelope and insufficient insulation can have very low winter indoor mean temperature (14.9 °C in Table 2) and quite high mean relative humidity (70% in Table 6), and a large fluctuation in indoor air temperature and relative humidity during the winter. The indoor mean minimum temperature can go down to 6 °C and the indoor mean maximum relative humidity can go up to 85%. During school hours, there were only 51%, 27% and 7% of time when indoor air temperatures were greater than or equal to 16 °C (the minimum threshold of indoor temperature required for limiting respiratory infections [3,4]), 18 °C and 20 °C (the minimum indoor air temperature required by WHO [1,2]), respectively. During the winter there was only 2.7% of time when indoor relative humidity was in the range of 40% to 60% (the optimal range of relative humidity for indoor air quality and minimising the indirect health effects of relative humidity such as bacteria, viruses, fungi, mites, respiratory rhinitis and asthma, and chemical interactions [26]). Classroom 1 (prefab) had Classroom 1 (prefab) with insufficient insulation in its envelope had very poor indoor thermal environment related to students’ thermal comfort and health. In New Zealand, those old school buildings should be fully retrofitted with sufficient insulation according to the current building codes [38,47] to improve the winter indoor thermal environment related to students’ thermal comfort and health.

Based on a comparison of the field-study data between Classroom 1 (prefab) and Classroom 2 (retrofitted) in Table 3 and Table 6, the indoor mean temperature of Classroom 2 (retrofit) was 3.6 °C higher than that of Classroom 1 (prefab). Classroom 2 (retrofit) had 34%, 43% and 41% more time than Classroom 1 (prefab) at indoor air temperatures greater than or equal to 16 °C, 18 °C and 20 °C, respectively, during winter school hours. During the winter, Classroom 2 (retrofit) had 42.8% more time than Classroom 1 (prefab) when indoor relative humidity was in the optimal range of 40% to 60%. Retrofitting an old school with sufficient insulation according to the current building codes [38,47] can significantly improve winter indoor thermal environment related to students’ thermal comfort and health. Based on the field-study data of Classroom 2 (retrofitted), this study identifies that a disadvantage of the conventional school building, with a timber structure, lightweight building envelope and sufficient insulation in the local climate with a mild and humid winter, is that the sufficient insulation in the building envelope can increase winter indoor mean air temperature and decrease indoor mean relative humidity, but cannot reduce the large fluctuation of winter indoor air temperatures and relative humidity, which still results in very low indoor air temperatures, e.g., the indoor mean minimum temperature can go down to 8.5 °C (see Table 3) and very high relative humidity, e.g., the indoor mean maximum relative humidity can go up to 84% (see Table 6). Winter daily indoor minimum air temperatures occur during the early morning, just before school hours. The very low indoor air temperature of Classroom 2 (retrofitted) is a challenge for maintaining indoor thermal comfort in the morning of school hours, which takes time to heat the space up to the comfort level temperature, e.g., 18 °C. According to winter indoor hourly mean air temperature of Classroom 2 (retrofit) in Figure 10, it took over four hours to rise indoor hourly mean air temperature from 14.1 °C (at 6 a.m.) to 18 °C (at 10 a.m.), which not only negatively impacts students’ thermal comfort and health but also potentially costs more space-heating energy. Increasing insulation in the building envelope without thermal mass can increase indoor mean air temperature and decrease indoor relative humidity but cannot reduce the fluctuation of indoor air temperature and relative humidity during the winter. 

Based on the winter field-study data of Classroom 3 (thermal mass) and Classroom 2 (retrofitted) in Table 2, Classroom 3 with thermal mass has 31%, 34% and 9% more time than Classroom 2 without thermal mass when indoor air temperatures were greater than or equal to 16 °C (the minimum threshold of indoor temperature required for limiting respiratory infections [3,4]), 18 °C and 20 °C (the minimum indoor air temperature required by WHO [1,2]), respectively. For Classroom 3 (thermal mass), there was only 6% of time in winter when indoor air temperatures were lower than 16 °C. For Classroom 2 (retrofit), there was 37%, 15% and 3% of time in winter when indoor air temperatures were lower than 16 °C, 14 °C and 12 °C respectively. These very low indoor air temperatures can negatively impact occupants’ health [3,4,5,6,7]. Classroom 3 (thermal mass) with thermal mass has 21.4% more time than Classroom 2 (retrofit) without thermal mass when indoor relative humidity was in the optimal range of 40% to 60%. Classroom 3 (thermal mass) had 17%, 2% and 1% less time than Classroom 2 (retrofit) at indoor relative humidity greater than or equal to 60%, 75% and 80%, respectively (60% of relative humidity is the threshold of mould survival and growth conditions, 75–80% of relative humidity are required for dust mites to thrive and 80% of relative humidity is the threshold of mould germination [41,42,43,44]). According to winter hourly mean temperatures and relative humidity of Classroom 3 (thermal mass) in Figure 10 and Figure 11, winter indoor hourly mean temperatures of Classroom 3 (thermal mass) are more stable and significantly higher than those of Classroom 2 (retrofit) during the late afternoon, evening, night and early morning, and winter hourly mean temperature of Classroom 3 (thermal mass) is always higher than 18 °C or close to 18 °C (the minimum winter hourly mean temperature is 17.8 °C). Winter indoor hourly mean relative humidity of Classroom 3 (thermal Mass) was more stable and lower than Classroom 2 (retrofit) and was always in the healthy range 50% and 60% (see Figure 11). Classroom 3 with thermal mass has a significantly better winter indoor thermal environment related to students’ thermal comfort and health than Classroom 2 without thermal mass. Adding thermal mass in the building envelope can not only increase indoor mean air temperature and decrease indoor mean relative humidity, but also reduce fluctuation of indoor air temperature and relative humidity. This can reduce the incidence of very low indoor air temperatures and very high indoor relative humidity, and significantly improve the winter indoor thermal environment related to students’ thermal comfort and health. Adding thermal mass in the local school building should be considered as a strategy or a guideline to improve the winter indoor thermal environment related to students’ thermal comfort and health for future school design and development in a temperate climate with mild and humid winter.

## Figures and Tables

**Table 1:** Construction elements of the three classrooms.

Building Elements	Classroom 1 (Prefab)	Classroom 2 (Retrofit)	Classroom 3 (Thermal Mass)
Structure	Timber	Timber	Precast concrete
Roof elements	Iron roofing	Steel roofing	Long run steel roofing
	R1.9 fibre insulation	45 mm roof battens	Roofing underlay paper
		Building wrap	90 mm × 45 mm high timber on top of purlins with R1.0 polyester insulation
		25 mm ceiling batten 150 mm rafters with R2.9 polyester insulation	300 mm purlin zone with steel DHS and R2.2 polyester insulation
		13 mm Plaster board	13 mm sound rated plasterboard
			200 mm air gap
			Suspended ceiling tile system
Wall elements	Timber/plasterboard	Fundamax high pressure laminate cladding	N. E. W. wall elements:
	R1.5 fibre insulation	25 mm cavity battens	70 mm facia concrete panel
		building wrap	40 mm XPS rigid insulation
		90 mm framing with R2.2 polyester	Internal 150 mm concrete
			S. wall elements:
			Fundamax laminate panels or weatherboards
			20 mm cavity batten
			building wrap
			140 mm concrete framing with R 2.2 insulation
			13 mm plaster board
Floor elements	Old carpet	Nylon carpet	Floor concrete slab with in-situ heating pipes
	wood floorboards	20 mm hardwood floorboards	40 mm XPS rigid insulation boards under slab
	R0.9 polyester insulation	Floor 150 mm joists with R2.4 (150 mm polyester)	Concrete floor
Glazing	Single glazing	Double glazing	Double glazing

**Table 2:** Indoor temperatures and percentages of time related to different temperature ranges throughout winter (24 h per day).

Classrooms	Classroom 1 (Prefab)			Classroom 2 (Retrofit)			Classroom 3 (Thermal Mass)			
	Floor	Ceiling	Mean	Floor	Ceiling	Mean	Floor	Ceiling	Mean	Outdoor
Mean T (°C)	14.6	15.2	14.9	17.0	17.5	17.3	18.9	19.2	19.0	12.2
Max T (°C)	20.8	24.2	22.0	29.1	28.2	27.4	24.0	25.5	24.2	20.6
Min T (°C)	6.7	5.4	6.0	8.7	8.3	8.5	14.5	14.2	14.3	1.9
Fluctuation	14.1	18.8	16.0	20.4	19.9	18.9	9.5	11.3	9.9	18.7
Time T ≥ 16 °C	31%	42%	37%	61%	65%	63%	94%	93%	94%	11%
Time T ≥ 18 °C	9%	22%	15%	36%	42%	40%	73%	75%	74%	2%
Time T ≥ 20 °C	0%	8%	3%	16%	25%	21%	26%	32%	30%	0%
Time T ≥ 22 °C	0%	2%	0%	5%	11%	7%	1%	6%	3%	0%
Time T ≥ 24 °C	0%	0%	0%	1%	3%	1%	0%	0%	0%	0%
Time T ≥ 26 °C	0%	0%	0%	0%	0%	0%	0%	0%	0%	0%

**Table 3:** Indoor temperatures and percentages of time related to different temperature ranges during winter school hours (from 8:30 a.m. to 3:30 p.m.).

Classrooms	Classroom 1 (Prefab)			Classroom 2 (Retrofit)			Classroom 3 (Thermal Mass)			
	Floor	Ceiling	Mean	Floor	Ceiling	Mean	Floor	Ceiling	Mean	Outdoor
Mean T (°C)	14.9	16.6	15.8	18.6	20.1	19.4	19.4	19.9	19.6	14.3
Max T (°C)	20.8	24.2	22.0	29.1	28.2	27.4	24.0	25.5	24.2	20.6
Min T (°C)	6.7	5.4	6.0	8.7	8.3	8.5	14.6	14.4	14.5	2.6
Fluctuation	14.1	18.8	16.0	20.4	19.9	18.9	9.4	11.1	9.7	18.0
Time T ≥ 16 °C	38%	60%	51%	79%	87%	85%	95%	95%	95%	27%
Time T ≥ 18 °C	13%	41%	27%	61%	77%	70%	78%	83%	80%	7%
Time T ≥ 20 °C	0%	18%	7%	35%	59%	48%	38%	51%	46%	0%
Time T ≥ 22 °C	0%	4%	0%	13%	31%	20%	4%	16%	9%	0%
Time T ≥ 24 °C	0%	0%	0%	3%	8%	4%	0%	1%	0%	0%
Time T ≥ 26 °C	0%	0%	0%	0%	1%	1%	0%	0%	0%	0%

**Table 4:** Indoor temperatures and percentages of time related to different temperature ranges during winter nights (from 7 p.m. to 7 a.m.).

Classrooms	Classroom 1 (Prefab)			Classroom 2 (Retrofit)			Classroom 3 (Thermal Mass)			
	Floor	Ceiling	Mean	Floor	Ceiling	Mean	Floor	Ceiling	Mean	Outdoor
Mean T (°C)	14.2	14.0	14.1	15.8	15.8	15.8	18.6	18.6	18.6	10.7
Max T (°C)	18.9	20.1	19.5	22.3	23.9	23.1	21.5	22.0	21.7	17.7
Min T (°C)	7.3	6.0	6.7	9.1	8.8	9.0	14.7	14.4	14.6	2.0
Fluctuation	11.6	14.1	12.8	13.2	15.1	14.1	6.8	7.6	7.1	15.7
Time T ≥ 16 °C	21%	24%	23%	47%	46%	46%	93%	92%	93%	1%
Time T ≥ 18 °C	4%	6%	5%	16%	16%	16%	70%	70%	70%	0%
Time T ≥ 20 °C	0%	0%	0%	2%	4%	3%	16%	18%	17%	0%
Time T ≥ 22 °C	0%	0%	0%	0%	1%	0%	0%	0%	0%	0%
Time T ≥ 24 °C	0%	0%	0%	0%	0%	0%	0%	0%	0%	0%
Time T ≥ 26 °C	0%	0%	0%	0%	0%	0%	0%	0%	0%	0%

**Table 5:** Indoor temperature and pecentages of time related to different low temperature ranges throughout winter (24 h per day).

Classrooms	Classroom 1 (Prefab)			Classroom 2 (Retrofit)			Classroom 3 (Thermal Mass)			
	Floor	Ceiling	Mean	Floor	Ceiling	Mean	Floor	Ceiling	Mean	Outdoor
Mean T (°C)	14.6	15.2	14.9	17.0	17.5	17.3	18.9	19.2	19.0	12.2
Max T (°C)	20.8	24.2	22.0	29.1	28.2	27.4	24.0	25.5	24.2	20.6
Min T (°C)	6.7	5.4	6.0	8.7	8.3	8.5	14.5	14.2	14.3	1.9
Fluctuation	14.1	18.8	16.0	20.4	19.9	18.9	9.5	11.3	9.9	18.7
Time T < 9 °C	2%	3%	2%	0%	0%	0%	0%	0%	0%	18%
Time T < 10 °C	4%	7%	6%	0%	0%	0%	0%	0%	0%	25%
Time T < 12 °C	16%	17%	17%	3%	3%	3%	0%	0%	0%	46%
Time T < 14 °C	38%	35%	36%	16%	15%	15%	0%	0%	0%	70%
Time T < 16 °C	69%	58%	63%	39%	35%	37%	6%	7%	6%	89%

**Table 6:** Indoor mean relative humidity and pecentage of time related to different relative humidity ranges throughout winter (24 h per day).

Classrooms	Classroom 1 (Prefab)			Classroom 2 (Retrofit)			Classroom 3 (Thermal Mass)			
	Floor	Ceiling	Mean	Floor	Ceiling	Mean	Floor	Ceiling	Mean	Outdoor
Mean RH (%)	70	70	70	61	59	60	58	57	58	80
Max RH (%)	84	85	84	84	83	84	77	76	76	99
Min RH (%)	51	52	53	28	32	30	37	33	35	35
Time RH ≥ 40%	100%	100%	100%	99%	98%	99%	100%	100%	100%	100%
Time RH ≥ 50%	100%	100%	100%	91%	84%	88%	90%	89%	90%	98%
Time RH ≥ 60%	97%	96%	97%	57%	49%	53%	33%	31%	32%	93%
Time RH ≥ 70%	53%	53%	53%	11%	8%	9%	4%	3%	3%	78%
Time RH ≥ 75%	16%	15%	14%	2%	2%	2%	1%	0%	0%	69%
Time RH ≥ 80%	1%	2%	1%	1%	1%	1%	0%	0%	0%	58%
Time RH ≥ 85%	0%	0%	0%	0%	0%	0%	0%	0%	0%	44%
Time RH ≥ 90%	0%	0%	0%	0%	0%	0%	0%	0%	0%	26%
Time RH ≥ 100%	0%	0%	0%	0%	0%	0%	0%	0%	0%	0%
Time 40% ≤ RH ≤ 60%	3.2%	3.8%	2.7%	41.9%	49.1%	46.0%	66.5%	68.6%	67.4%	7.5%
